# Admission serum sodium and potassium levels predict survival among critically ill patients with acute kidney injury: a cohort study

**DOI:** 10.1186/s12882-019-1505-9

**Published:** 2019-08-08

**Authors:** Xu-ping Gao, Chen-fei Zheng, Min-qi Liao, Hong He, Yan-hua Liu, Chun-xia Jing, Fang-fang Zeng, Qing-shan Chen

**Affiliations:** 1 Department of Epidemiology, School of Medicine, Jinan University, No.601 Huangpu Road West, Guangzhou, 510630 Guangdong China; 2 Department of Nephrology, the First Affiliated Hospital of Wenzhou Medical University, Wenzhou, 325000 China; 3 Health Care and Physical Examination Center, The First Affiliated Hospital, Sun Yat-sen University, Guangzhou, 510080 China; 4 The First Affiliated Hospital of Zhengzhou University, No. 1 East Jianshe Road, Zhengzhou, 450052 Henan China

**Keywords:** Acute kidney injury, Serum sodium, Serum potassium, Prognosis, Mortality

## Abstract

**Background:**

Patients suffering from acute kidney injury (AKI) were associated with impaired sodium and potassium homeostasis. We aimed to investigate how admission serum sodium and potassium independently and jointly modified adverse clinical outcomes among AKI patients.

**Methods:**

Patient data were extracted from the Multiparameter Intelligent Monitoring in Intensive Care Database III. Participants were categorized into three groups according to admission serum sodium and potassium, and the cut-off values were determined using smooth curve fitting. The primary outcome was 90-day mortality in the intensive care unit (ICU). Cox proportional hazards models were used to evaluate the prognostic effects of admission serum sodium and potassium levels.

**Results:**

We included 13,621 ICU patients with AKI (mean age: 65.3 years; males: 55.4%). The middle category of admission serum sodium and potassium levels were 136.0–144.9 mmol/L and 3.7–4.7 mmol/L through fitting smooth curve. In multivariable Cox models, compared with the middle category, patients with hyponatremia or hypernatremia were associated with excess mortality and the HRs and its 95%CIs were 1.38 (1.27, 1.50) and 1.56 (1.36, 1.79), and patients with either hypokalemia or hyperkalemia were associated with excess mortality and the hazard ratios (HRs) and its 95% confidential intervals (95% CIs) were 1.12 (1.02, 1.24) and 1.25 (1.14, 1.36), respectively. Significant interactions were observed between admission serum sodium and potassium levels (P interaction = 0.001), with a higher serum potassium level associated with increased risk of 90-day mortality among patients with hyponatremia, whereas the effects of higher sodium level on prognostic effects of potassium were subtle.

**Conclusions:**

Admission serum sodium and potassium were associated with survival in a U-shaped pattern among patients with AKI, and hyperkalemia predict a worse clinical outcome among patients with hyponatremia.

**Electronic supplementary material:**

The online version of this article (10.1186/s12882-019-1505-9) contains supplementary material, which is available to authorized users.

## Introduction

Acute kidney injury (AKI) is a common complication observed among hospitalised patients, especially in the intensive care unit (ICU) [1], which account for 37% of patients treated in ICU [2, 3]. AKI has been supposed to be associated with short-term and long-term in-hospital mortality in various clinical settings [4–6]. In the presence of AKI, the mortality rate of patient increases to as high as 60–70%, especially within 1 year after ICU admission [6, 7]. Moreover, survivors of AKI are at increased risk of kidney function decline and end-stage renal disease [8, 9]. Therefore, identifying clinical predictors of mortality in AKI may help to improve its prognosis.

The kidneys play a central role in sodium and potassium homeostasis, and its function decline leads to electrolyte disorder. Abnormal sodium levels (hyponatremia or hypernatremia) may often be accompanied by hypokalemia or hyperkalemia. In hospitalised patients, there are numerous factors, including underlying comorbidities (e.g., heart diseases and cancers) and medication use (e.g., angiotensin-converting enzyme inhibitors (ACEIs) and angiotensin II receptor blockers (ARBs)), that might affect the serum sodium and potassium levels [10, 11]. Recently, both below and above the clinically accepted normal range of admission serum potassium was found to associate with excess mortality in a U-shaped relationship among hospitalised patients with several diseases, such as hypertension, heart failure, chronic kidney disease, or diabetes [12–14]. Correspondingly, a similar picture has emerged for serum sodium levels in hospitalised patients with chronic kidney disease [15] or undergoing major orthopedic surgery [16]. However, the independent or synergistic prognostic effects of abnormal serum sodium and potassium levels remain less well studied among hospitalised AKI patients [15]. In addition, normal ranges of serum sodium and potassium levels applicable to AKI patients are still unknown [16]. Therefore, identifying normal ranges of serum sodium and potassium levels with clinical significance is an important issue for clinicians to make decision when electrolyte imbalance occurs among AKI patients [17].

Using data from the Multiparameter Intelligent Monitoring in Intensive Care Database III version 1.3 (MIMIC-III, v1.3), we aimed to explore potential prognostic effects and their normal ranges of baseline serum sodium and potassium among AKI patients.

## Materials and methods

The study was conducted according to STROBE (Strengthening the reporting of observational studies in epidemiology) guidelines (Additional file 1: Table S1) [18].

### Data source

Present study was based on the publicly and freely available database known as MIMIC-III (version 1.3). This database was approved by the Institutional Review Boards of Beth Israel Deaconess Medical Center (Boston, MA, USA) and the Massachusetts Institute of Technology (Cambridge, MA, USA). Requirement for individual patient consent was waived because the project did not impact clinical care and all protected health information was deidentified [19]. The authors had got access to the database after completion of the recognized course “Protecting Human Research Participants” (certification number: 1605699 and 29,072,172).

### Population selection criteria

Critically ill adult patients were included in our study. Patients’ inclusion criteria were: 1) patients who were older than 18 years old; 2) length of stay in the ICU > 2 days; 3) patients suffered AKI. AKI was determined based on the Kidney Disease: Improving Global Outcomes (KDIGO) classification [20]: serum creatinine (SCr) improved more than 1.5 times within the prior 7 days of admission; SCr increased 0.3 mg/dl within 48-h period; and urine output lower than 0.5 ml/kg/h per 6-h. The baseline serum creatinine was defined as the available SCr value prior to hospitalization or the first measured value during hospitalization following the recommendation of the International Club of Ascites [21]. Patients were excluded from our study if > 5% of their individual data were missing. Finally, patients with extreme outliers of admission serum sodium and potassium levels in the lowest 0.10th (≤113 mmol/L and ≤ 2.2 mmol/L, respectively) and highest 99.9th (≥169.7 mmol/L and ≥ 9.4 mmol/L, respectively) percentiles were excluded.

### Data extraction

The MIMIC-III data were collected from 2001 to 2012 at Beth Israel Deaconess Medical Center. Data extraction was performed by using Structured Query Language (SQL) with MySQL tools (version 5.6.24), and following baseline characteristics within the first 24 h after patient admission were extracted: 1) patient identifiers; 2) demographic parameters: age, and gender; 3) clinical parameters: urine output, renal replacement therapy (RRT), follow-up data (start at patient’s admission, and followed for at least 3 months), death data (obtained from Social Security Death Index records from the U.S. government). The outcomes of our study were defined as 30-day and 90-day all-cause mortality; 4) laboratory parameters: admission serum creatinine (SCr) levels, serum sodium levels, and serum potassium levels; 5) scoring systems: Glasgow Coma Scale (GCS) score and non-renal Sepsis Related Organ Failure Assessment (non-renal SOFA) score.

### Statistical analysis and modeling strategy

Baseline characteristics of included patients were grouped by cut-offs of admission serum sodium and potassium levels and are presented as frequency (percent) for categorical data and as mean (SD) or median (IQR) for continuous data. Comparisons of means between groups were performed using the the analysis of variance (ANOVA) for normally distributed variables and the Kruskal-Wallis test for non-normally distributed variables; the Chi-square test was used to analyze categorical variables. The Kaplan-Meier curves were carried out to explore the influence of admission serum sodium and potassium levels on the 90-day mortality as well as 30-day mortality.

To identify admission serum sodium and potassium cut-offs, we depicted 90-day mortality as hazard functions of serum sodium and potassium levels, and nonparametric estimates was conducted by R smoothHR package. Adjustments were made for all covariates. Based on this, we grouped low-risk serum sodium as 136.0–144.9 mmol/L and the remaining as < 136.0 mmol/L and ≥ 145.0 mmol/L low-risk serum potassium as 3.7–4.7 mmol/L and the remaining as < 3.7 mmol/L, and ≥ 4.8 mmol/L. We also grouped the patients into quintiles according to the distribution of serum sodium and potassium levels to estimate their associations with mortality. The proportional hazards assumption was examined by comparing plots of the cumulative hazard functions across exposure categories. No appreciable violations in the assumption were found. Cox proportional hazards models were used to analyze the independent or synergistic prognostic effects of abnormal admission serum sodium and potassium levels. The primary outcome was 90-day mortality, and the secondary outcome was 30-day mortality. The middle category or the third quintile group of admission serum sodium and potassium levels was treated as the reference group. We used two models to calculate hazard ratios (HRs) with their corresponding 95% confidence intervals (CIs). In model 1, covariates were adjusted only for age and sex; in model 2, we further adjusted for admission blood urea nitrogen, creatinine, urine output, GCS score, non-renal SOFA score, vasopressin use, ventilator use, cardiovascular diseases (CVDs, including cardiac arrhythmias, pulmonary circulation, and valvular disease), chronic pulmonary disease, deficiency anemias, liver disease, tumor related diseases (including lymphoma, metastatic cancer, and solid tumor). All of these covariates were introduced using the forward stepwise method. Multiplicative interactions were estimated by adding interaction terms according to the likelihood ratio test. We also analyzed the prognostic effects of baseline serum sodium levels across different potassium strata to assess their joint effects.

We further conducted stratification analyses to examine whether the effect of admission serum sodium or potassium differed across various subgroups classified by age (< 65 vs. ≥65 years), sex (male vs. female), SCr (< 1.50 vs. ≥1.50 mg/dl), urine output (< 1120 vs. ≥1120 ml/24 h), vasopressin use (yes or no), and RRT (yes or no). We also performed sensitivity analyses to test the robustness of our results through excluding participants complicated with CVDs, chronic pulmonary disease, deficiency anemias, liver disease, or tumor related diseases.

Statistical analyses were performed using R software (version 3.5.1) and SPSS software (version 21.0). The *P* value of < 0.05 was considered significant.

## Results

### Subject characteristics

Patient records from 14,354 AKI patients were initially extracted from the MIMIC-III database. After excluding 669 patients stayed in the hospital within 2 days and 64 patients with extreme outliers of admission serum sodium and potassium levels, 13,621 eligible subjects were finally included in the present study. The subjects included 7543 men and 6078 women, and were at the age from 18 to 88 years, with a mean age of 65.3 ± 15.7 years. Of these subjects, 8051 (59.1%) patients were recruited from the medical ICU, and 5434 (40.9%) patients were recruited from the surgical ICU.

Overall means (SD) for admission serum sodium and potassium levels were 137.8 (5.2) mmol/L and 4.36 (0.85) mmol/L, respectively. 3678 (27.0%) patients were in the low sodium group (< 136.0 mmol/L), 9161 (67.3%) patients in the middle sodium group (136.0–144.9 mmol/L), and 782 (5.7%) patients in the high sodium group (≥145.0 mmol/L). 2259 (16.6%) patients were grouped into the low potassium group (< 3.7 mmol/L), 8032 (59.0%) patients in the middle potassium group (3.7–4.7 mmol/L), and 3330 (24.4%) patients in the high potassium group (≥4.8 mmol/L).

**Fig. 1:**
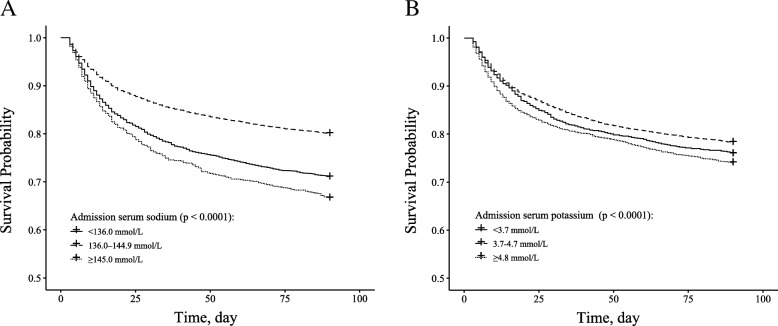
Kaplan–Meier survival curves for 90-day all-cause mortality (**a**: admission serum potassium; **b**: admission serum potassium)

The summary of the baseline characteristic of the study population are provided in Additional file 1: Table S2 and S3. A total of 2157 30-day and 3154 90-day deaths occurred during follow-up. Survival curves for 90-day all-cause mortality across categories of admission serum sodium and potassium are presented in Fig. 1. There was a significant difference in survival probability across categories of both sodium and potassium (both *P* < 0.0001).

**Fig. 2:**
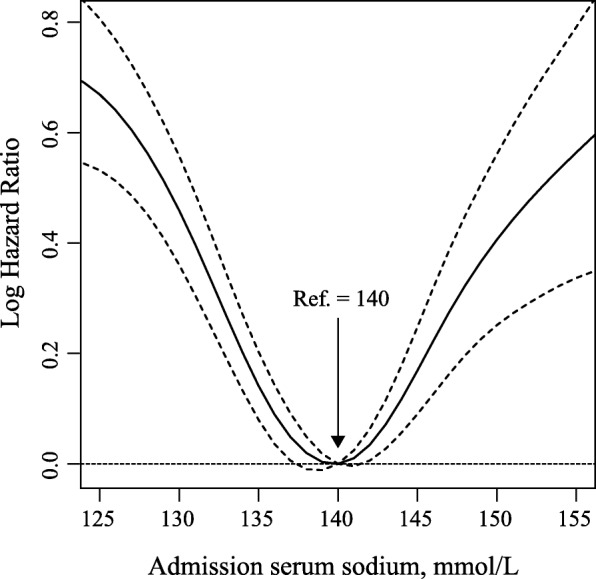
Nonparametric estimates of 90-day all-cause mortality on admission serum sodium among patients with AKI (log hazard ratio, with 95% confidence limits, adjusted for age and gender)

**Fig. 3:**
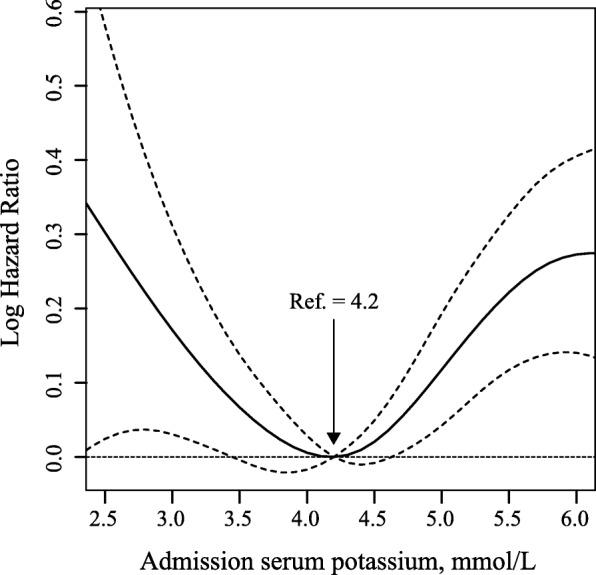
Nonparametric estimates of 90-day all-cause mortality on admission serum potassium among patients with AKI (log hazard ratio, with 95% confidence limits, adjusted for age and gender)

Association between admission serum sodium and potassium levels and 90-day and 30-day survival. After adjustment for age and gender, U-shaped associations were observed for both sodium and potassium levels with 90-day mortality (Figs. 2 and 3). With further adjustments made for other covariates, significant associations slightly attenuated but admission remained. For serum sodium, compared with reference group (136.0–144.9 mmol/L), patients with hyponatremia (< 136.0 mmol/L) or hypernatremia (≥145.0 mmol/L) were associated with excess mortality and the HRs and its 95%CIs were 1.38 (1.27, 1.50) and 1.56 (1.36, 1.79). For serum potassium, compared with reference group (3.7–4.7 mmol/L), patients with hypokalemia (< 3.7 mmol/L) or hyperkalemia (≥4.8 mmol/L) were associated with excess mortality and the HRs and its 95%CIs were 1.12 (1.02, 1.24) and 1.25 (1.14, 1.36), respectively. Similar associations were also observed when using quintiles as cut-offs or 30-day deaths as outcome for both serum sodium and potassium levels (Tables 1 and 2).

**Table 1:** Relationship between admission serum sodium level and mortality among critically ill patients with acute kidney injury

Serum sodium level, mmol/L	No. of patients/deaths	Model 1 ^a^		Model 2 ^b^	
		HR (95% CIs)	*P* value	HR (95% CIs)	*P* value
90-Day all-cause mortality					
Fitted groups					
< 136.0	3678/1066	1.68 (1.55, 1.81)	< 0.001	1.38 (1.27, 1.50)	< 0.001
136.0–144.9	9161/1827	1.00		1.00	
≥ 145.0	782/261	1.76 (1.54, 2.01)	< 0.001	1.56 (1.36, 1.79)	< 0.001
Quintiles					
< 135.0	2910/880	2.00 (1.81, 2.20)	< 0.001	1.57 (1.41, 1.75)	< 0.001
135.0–138.9	4193/902	1.22 (1.10, 1.35)	< 0.001	1.16 (1.04, 1.28)	0.005
139.0–141.9	3914/722	1.00		1.00	
142.0–145.9	2063/459	1.25 (1.11, 1.40)	< 0.001	1.24 (1.10, 1.40)	< 0.001
≥ 146.0	541/191	2.05 (1.74, 2.41)	< 0.001	1.75 (1.48, 2.07)	< 0.001
30-Day all-cause mortality					
Fitted groups					
< 136.0	3678/747	1.70 (1.55, 1.86)	< 0.001	1.37 (1.24, 1.51)	< 0.001
136.0–144.9	9161/1226	1.00		1.00	
≥ 145.0	782/184	1.79 (1.53, 2.10)	< 0.001	1.51 (1.28, 1.78)	< 0.001
Quintiles					
< 135.0	2910/618	2.03 (1.80, 2.29)	< 0.001	1.55 (1.36, 1.77)	< 0.001
135.0–138.9	4193/623	1.25 (1.11, 1.41)	< 0.001	1.18 (1.05, 1.34)	0.008
139.0–141.9	3914/479	1.00		1.00	
142.0–145.9	2063/300	1.22 (1.05, 1.41)	0.008	1.20 (1.03, 1.39)	0.018
≥ 146.0	541/137	2.12 (1.75, 2.58)	< 0.001	1.68 (1.37, 2.05)	< 0.001

Abbreviations: *CI* confidence interval, *HR* hazard ratio

^a^ Models were derived from Cox proportional hazards regression models. Model 1 covariates were adjusted for age, and sex

^b^ Model 2 covariates were also adjusted for blood urea nitrogen, creatinine, urine output, Glasgow Coma Scale score, non-renal Sepsis Related Organ Failure Assessment score, vasopressin use, ventilator use, cardiovascular diseases, chronic pulmonary disease, deficiency anemias, liver disease, tumor related diseases

**Table 2:** Relationship between admission serum potassium level and mortality among critically ill patients with acute kidney injury

Serum potassium level, mmol/L	No. of patients/deaths	Model 1 ^a^		Model 2 ^b^	
		HR (95% CIs)	*P* value	HR (95% CIs)	*P* value
90-Day all-cause mortality					
Fitted groups					
< 3.7	2259/544	1.20 (1.09, 1.33)	< 0.001	1.12 (1.02, 1.24)	0.023
3.7–4.7	8032/1745	1.00		1.00	
≥ 4.8	3330/865	1.26 (1.16, 1.37)	< 0.001	1.25 (1.14, 1.36)	< 0.001
Quintiles					
< 3.5	1263/302	1.22 (1.07, 1.39)	0.003	1.08 (0.94, 1.24)	0.256
3.5–3.9	3235/726	1.07 (0.97, 1.18)	0.168	1.05 (0.95, 1.16)	0.361
4.0–4.4	4077/889	1.00		1.00	
4.5–4.9	2579/587	1.05 (0.94, 1.17)	0.379	1.05 (0.94, 1.17)	0.423
≥ 5.0	2467/650	1.31 (1.18, 1.45)	< 0.001	1.27 (1.14, 1.42)	< 0.001
30-Day all-cause mortality					
Fitted groups					
< 3.7	2259/381	1.24 (1.10, 1.39)	< 0.001	1.15 (1.02, 1.30)	0.022
3.7–4.7	8032/1164	1.00		1.00	
≥ 4.8	3330/612	1.32 (1.20, 1.46)	< 0.001	1.32 (1.19, 1.47)	< 0.001
Quintiles					
< 3.5	1263/215	1.30 (1.11, 1.52)	0.001	1.15 (0.97, 1.35)	0.103
3.5–3.9	3235/504	1.13 (1.00, 1.27)	0.049	1.09 (0.96, 1.23)	0.181
4.0–4.4	4077/579	1.00		1.00	
4.5–4.9	2579/395	1.08 (0.95, 1.22)	0.270	1.08 (0.95, 1.23)	0.254
≥ 5.0	2467/464	1.41 (1.25, 1.59)	< 0.001	1.37 (1.20, 1.56)	< 0.001

Abbreviations: *CI* confidence interval, *HR* hazard ratio

^a^ Models were derived from Cox proportional hazards regression models. Model 1 covariates were adjusted for age, and sex

^b^ Model 2 covariates were also adjusted for blood urea nitrogen, creatinine, urine output, Glasgow Coma Scale score, non-renal Sepsis Related Organ Failure Assessment score, vasopressin use, ventilator use, cardiovascular diseases, chronic pulmonary disease, deficiency anemias, liver disease, tumor related diseases

**Fig. 4:**
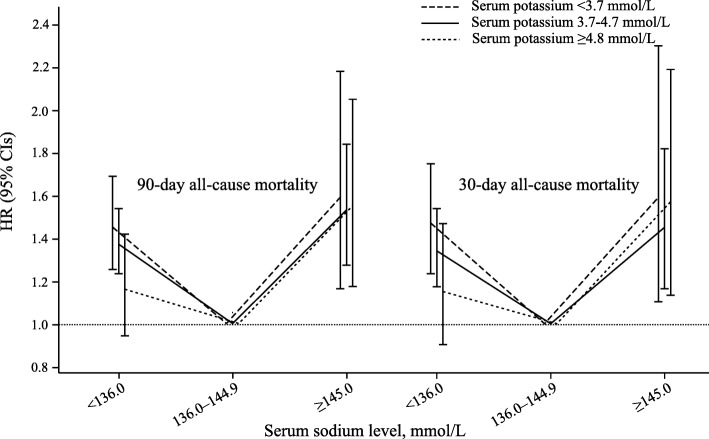
90-day and 30-day all-cause mortality according to admission serum sodium levels across different potassium strata. Abbreviations: CI, confidence interval; HRs, hazard ratios

Significant interactions were observed between admission serum sodium and potassium levels on both 90-day mortality (P interaction = 0.001) and 30-day mortality (P interaction = 0.007). Among patients with lower serum sodium level (< 136.0 mmol/L), a higher serum potassium level was associated with increased risk of 90-day mortality (HR for potassium ≥4.8 vs. < 3.7 mmol/L: 1.45 vs. 1.16) and 30-day mortality (HR for potassium ≥4.8 vs. < 3.7 mmol/L: 1.47 vs. 1.15), whereas the effects of higher sodium level on prognostic effects of potassium were subtle (Fig. 4 and Additional file 1: Table S4).

### Subgroup analyses

In the subgroup analyses for serum sodium, significant interactions were found for stratification according to SCr levels (P interaction: 0.005 for 90-day mortality and 0.027 for 30-day mortality). Patients with lower SCr have higher risk of mortality than patients with higher SCr for hyponatremia (Table 3 and Additional file 1: Table S5).

**Table 3:** Subgroup analysis of the associations between 90-day all-cause mortality and the admission serum sodium level

	No. of patients/deaths	Admission serum sodium level, mmol/L			P for interaction
		< 136.0	136.0–144.9	≥145.0	
Age, years					
< 65	5772/956	1.36 (1.18, 1.57)	1.00	1.64 (1.25, 2.15)	0.553
≥ 65	7687/2139	1.41 (1.28, 1.56)	1.00	1.52 (1.29, 1.78)	
Sex					
Male	7543/1766	1.38 (1.24, 1.54)	1.00	1.53 (1.27, 1.86)	0.333
Female	6078/1388	1.35 (1.19, 1.53)	1.00	1.62 (1.33, 1.98)	
Creatinine, mg/dl					
< 1.50	6295/1345	1.55 (1.37, 1.76)	1.00	1.27 (0.98, 1.63)	0.005
≥ 1.50	7324/1808	1.24 (1.11, 1.39)	1.00	1.59 (1.34, 1.88)	
Urine output, ml/24 h					
< 1120	6487/1943	1.31 (1.19, 1.45)	1.00	1.32 (1.11, 1.56)	0.136
≥ 1120	6520/1069	1.33 (1.16, 1.53)	1.00	1.71 (1.35, 2.17)	
Vasopressin use					
Yes	5907/1728	1.33 (1.19, 1.48)	1.00	1.49 (1.22, 1.83)	0.258
No	7714/1426	1.42 (1.26, 1.61)	1.00	1.55 (1.28, 1.87)	
Renal replacement therapy					
Yes	365/177	1.25 (0.91, 1.72)	1.00	1.10 (0.51, 2.40)	0.683
No	13,265/2977	1.38 (1.27, 1.50)	1.00	1.58 (1.37, 1.82)	

Hazard ratio (95% CI): from Cox proportional hazards regression models. Covariates adjusted for: see model 2 in Table 1

For serum potassium, the risk of 90-day mortality was similar for most strata (P ranged from 0.222 to 0.926), with the exception of significantly stronger associations among males than females for hyperkalemia (HR for male vs. female: 1.34 vs. 1.14; P interaction: 0.043) (Table 4). Similar finding was observed for 30-day mortality (Additional file 1: Table S6).

**Table 4:** Subgroup analysis of the associations between 90-day all-cause mortality and the admission serum potassium level

	No. of patients/deaths	Admission serum potassium level, mmol/L			P for interaction
		< 3.7	3.7–4.7	≥4.8	
Age, years					
< 65	5772/956	1.06 (0.89, 1.26)	1.00	1.34 (1.14, 1.57)	0.790
≥ 65	7687/2139	1.13 (1.00, 1.28)	1.00	1.21 (1.09, 1.34)	
Sex					
Male	7543/1766	1.13 (0.98, 1.31)	1.00	1.34 (1.20, 1.50)	0.043
Female	6078/1388	1.11 (0.97, 1.28)	1.00	1.14 (0.99, 1.31)	
Creatinine, mg/dl					
< 1.50	6295/1345	1.07 (0.93, 1.24)	1.00	1.33 (1.15, 1.54)	0.401
≥ 1.50	7324/1808	1.13 (0.98, 1.30)	1.00	1.20 (1.08, 1.34)	
Urine output, ml/24 h					
< 1120	6487/1943	1.08 (0.96, 1.23)	1.00	1.23 (1.11, 1.37)	0.222
≥ 1120	6520/1069	1.11 (0.94, 1.31)	1.00	1.23 (1.06, 1.43)	
Vasopressin use					
Yes	5907/1728	1.15 (1.01, 1.32)	1.00	1.34 (1.19, 1.51)	0.265
No	7714/1426	1.07 (0.92, 1.24)	1.00	1.13 (0.99. 1.28)	
Renal replacement therapy					
Yes	365/177	1.62 (1.03, 2.54)	1.00	1.58 (1.12, 2.23)	0.926
No	13,265/2977	1.11 (1.00, 1.23)	1.00	1.22 (1.12, 1.34)	

Hazard ratio (95% CI): from Cox proportional hazards regression models. Covariates adjusted for: see model 2 in Table 1

After excluding participants complicated with CVDs, chronic pulmonary disease, deficiency anemias, liver disease, or tumor related diseases, the associations between sodium or potassium and mortality (30-day or 90-day) were persisted (data not tabulated).

## Discussion

The present study had comprehensively explored the normal range and independent or synergistic prognostic effects of admission serum sodium and potassium levels among AKI patients. The normal range of serum sodium and potassium levels were 136.0–144.9 mmol/L and 3.7–4.7 mmol/L, respectively, through fitting smooth curve. We found that both lower and higher admission serum sodium or potassium levels were associated with excess mortality. Across different sodium strata, a higher admission serum potassium level was associated with increased risk of mortality among patients with lower sodium levels.

Normal ranges of serum sodium and potassium levels using in clinical sittings are determined mainly based on healthy subjects, whether these values are applicable to AKI patients with complex comorbidities is still unknown [16]. Our proposed cut-offs level for serum sodium (136.0–144.9 mmol/L) is generally in line with clinically accepted normal range (135.0–145.0 mmol/L); whereas for potassium, the clinically accepted normal range is 3.5–5.0 mmol/l, but we observed excess mortality out of a narrow range of 3.7–4.7 mmol/L. This indicates that the excess mortality in AKI patients with so-called normal potassium levels were considerable. This finding shows that the risk for mortality is higher not only for those with clinically abnormal potassium levels, but also for those with potassium levels in the normal range of 3.5–3.7 mmol/L and 4.8–5.0 mmol/L. In addition, significant interaction was observed between admission serum sodium and potassium levels, which attract the attention that clinicians should not only concern the abnormal serum sodium and potassium levels independently associated with the poor prognosis, but also take into account that interaction of them predicts higher risk of mortality.

The treatment and prevention of AKI remains a major challenge to the intensive care physicians [22]. Patients suffering from AKI were associated with impaired sodium and potassium homeostasis. Hyponatremia and hypernatremia, as well as hypokalemia and hyperkalemia, are common electrolyte abnormalities among critically ill patients [23–25]. No studies have investigated independently or simultaneously the prognostic role of both these electrolytes among patients with AKI. Similar with the findings from previous studies, abnormal serum sodium and potassium independently predicted an excess mortality among hospitalised patients with several diseases, such as hypertension, heart failure, chronic kidney disease, diabetes, or undergoing major orthopedic surgery [12–14, 26–28]. However, a strong interaction between serum potassium and sodium found in our study differs from the study by Polcwiartek C et al. [16], which suggested abnormal sodium is an important risk factor for mortality independent of potassium levels among patients with heart failure.

Significant interaction between serum sodium and potassium on mortality was observed in our study (P interaction: 0.001 for 90-day mortality, and 0.007 for 30-day mortality). Across different sodium strata, a higher admission serum potassium level was associated with increased risk of mortality among patients with lower sodium levels, whereas the effects of hypernatremia seemed to be independent of serum potassium. Hypernatremia has been poorly assessed in AKI, probably because according to previous report [29], more AKI patients suffered the hyponatremia (Na^+^ < 135 mmol/L) than hypernatremia (Na^+^ > 145 mmol/L) (36% vs 6%) when admitted into ICU. Similarly, the number of patients with hypernatremia was only 782 (5.7%) compared with 3678 (27.0%) patients with hyponatremia in our study. The significant lower number of patients among patients with hypernatremia suggests less statistical power to find a significant association. In addition, hypernatraemia cases more often had hypokalaemia [30], often complicated by hypertonic environment, also leads to high mortality. The poor prognosis of with hyponatremia, especially when combined with hyperkalemia, calls for action. The interaction might be explained by the Edelman equation: [*Na*^+^]_*plasma*_ = 1.03 (*Na*_*exhangeable*_ + *K*_*exchangeable*_)/Total body water ‐ 23.8, which shows that changes in the mass balance of Na^+^, K^+^ and H_2_O have an important impact on [Na^+^]_*plasma*_ [31]. We performed additional subgroup analyses and our findings suggest that the males with serum potassium ≥4.8 mmol/L have a higher risk of mortality than females (HRs with 95% CIs: 1.34 vs. 1.14 for male vs. female, P for interaction = 0.043), but independent of renal status. For serum sodium, patients with low SCr had a higher risk of mortality for hyponatremia. Low SCr value at admission has been suggested to be independently associated with increased in-hospital mortality in hospitalized patients [32]. Although diseases (e.g., CVDs) are common causes of impaired sodium and potassium homeostasis and associated with poor prognosis among AKI patients [12–14], but our sensitivity analysis of excluding diseases, such as CVDs, chronic pulmonary disease, deficiency anemias, liver disease, or tumor related diseases, did not change our conclusions.

This is the first study, to our knowledge, examining the association between serum sodium and potassium levels and mortality among AKI patients. The present study includes a large number of hospitalised patients allowing a relatively higher power to get precise exploration of the independent or joint prognostic effects of admission serum sodium or potassium levels.

There were also several limitations in this study. Firstly, it was a single-center retrospective analysis, we could be only able to establish an association, rather than a causal relationship. In addition, different results could be reached when using patient records from other centers. Therefore, subject selection bias cannot be ignored. Secondly, we only analyzed sodium or potassium levels at admission, these levels could be changed overtimes (e.g., developing certain comorbidities and initiating pharmacological therapy) and the association between the trajectory of serum potassium level and the outcome needs further confirmation. Thirdly, we only analyzed the influence of vasopressin use, but the use of diuretics (loop diuretics, thiazide diuretics, and mineralocorticoid receptor antagonists and renin-angiotensin system inhibitors (angiotensin-converting enzyme inhibitors, and angiotensinII-receptor blockers) were not included, and these treatments often disturb sodium or potassium homeostasis. Fourthly, although we attempted to account for a number of potential confounders in the multivariable analyses, it is plausible that other unidentified variables may have influenced the results. Finally, we used all-cause mortality, but not cause-specific death, was also a major limitation.

## Conclusions

This study indicated U-shaped associations between admission serum sodium or potassium levels and excess mortality in critically ill patients with AKI. Among patients with lower sodium levels, patients with higher admission serum potassium levels had increased risk of overall mortality. Our findings need to be confirmed by large prospective multicenter studies with longer follow-up.

## Additional file

Additional file 1: **Table S1.** STROBE Statement—checklist of items that should be included in reports of observational studies. **Table S2.** Baseline characteristics of the study population according to admission serum sodium level ^a^. **Table S3.** Baseline characteristics of the study population according to admission serum potassium level ^a^. **Table S4.** All-cause mortality according to admission serum sodium levels across admission serum potassium levels among critically ill patients with acute kidney injury. **Table S5.** Subgroup analysis of the associations between admission serum sodium level and 30-day all-cause mortality. **Table S6.** Subgroup analysis of the associations between admission serum potassium level and 30-day all-cause mortality. (DOCX 46 kb)

## Data Availability

This study was based on the database known as MIMIC-III (version 1.3). MIMIC-III is a large, freely-available database comprising deidentified health-related data associated with over forty thousand patients who stayed in critical care units of the Beth Israel Deaconess Medical Center between 2001 and 2012. The authors had got access to the database after completion of the recognized course “Protecting Human Research Participants” (certification number: 1605699 and 29072172). 10.1038/sdata.2016.35

## Abbreviations

ACEIs: Angiotensin-converting enzyme inhibitors
AKI: Acute kidney injury
ANOVA: Analysis of variance
ARBs: Angiotensin II receptor blockers
CI: Confidence interval
CKD: Chronic kidney disease
GCS: Glasgow Coma Scale
HR: Hazard ratio
ICU: Intensive care unit
IQR: Interquartile range
MIMIC-III: Multiparameter Intelligent Monitoring in Intensive Care Database III
RRT: Renal replacement therapy
SCr: Serum creatinine
SD: Standard deviation
SQL: Structured Query Language
